# Buildings and Fittings in the Older Hospitals

**Published:** 1898-01-01

**Authors:** 


### The Institutional Workshop.

### BUILDINGS AND FITTINGS IN THE OLDER HOSPITALS.

### A NECESSARY EXPENDITURE OF CAPITAL.

A visit to three or four hospitals on Christmas Day is an education in itself to the average resident in cities. He is thus able to see the marked differences which exist in the system pursued and to realise how great are sometimes the distinctions between systems of administration, although the cost per bed per annum may not greatly differ in any of the institutions visited. There is, for instance, the old type of hospital, where the furniture, the floors, the lighting, the fireplaces, and many other things are out of date and behind the times. We have always held that it is no excuse for a hospital committee in the present day to plead that the institution is no worse than it was when they took it over from their predecessors, or that the burden of annual maintenance is so great that they cannot find money to expend upon the fabric or the fittings so as to modernise their institution, and make it worthy of the hospital knowledge and excellence of to-day. On looking into institutions which make such excuses for the present neglected condition of the older portions of their buildings especially, one would seldom fail to find that the invested funds stood at an amount large enough to justify a considerable expenditure of those funds for the special purpose of renovating the hospital, and to thus free it from the discredit which justly attaches to any public building which is not properly kept up, and which is allowed to get into a condition less efficient than is essential to its utility. We venture to hope that one result of the Prince of Wales's Hospital Fund will be to remove blots of this kind from the hospitals of London, and that before long it will be impossible for any critic, however severe, to maintain that any of the great hospitals, or indeed that any hospital worthy of the name, is in a less efficient condition in every respect than it ought to be or than it would be if the management were intelligent and energetic.

Contrasts such as those just referred to were very noticeable in visiting first the Middlesex and then the London and Guy's Hospitals. Not so many years ago the internal condition of the buildings at Middlesex Hospital was shabby and unattractive. To-day, owing to the wisdom of its committee and the energy of its officers, Middlesex Hospital will compare in matters of fittings, furniture, and fabric with the most modern of hospitals, and we were amused to learn from the authorities that they had been severely criticised for extravagance because of the present excellent conditions. This amusement was due to the fact that the critics as usual were ignorant of the present position of Middlesex Hospital, which they ventured to condemn, for they were evidently unaware that, as compared with ten years ago, the invested funds show a considerable increase. At the London Hospital we are glad to learn that it is contemplated to modernise the entire institution by spending some £100,000 in the ensuing year upon the fabric, furniture and lighting. It would have been, indeed, a disappointment had so energetic a chairman as the Hon. Sydney Holland not initiated a wise policy of this kind, and one which, we make bold to say, will bring to the London Hospital increased public confidence and a large increase in public support. At Guy's Hospital it is imperatively necessary that the governors should spend at least £10,000 at once upon the old buildings and in renewing the furniture and fittings. We are quite aware that much has been done, especially of late, in this direction; but now that Guy's Hospital has been placed in funds to enable it to reopen the whole of its closed wards, it is essential that the Governors, remembering the large sums of money which the public have given to it during the last two years, should yield to public opinion and place the fabric of the institution in a presentable and attractive condition. To delay expenditure of this kind longer would be a grievous mistake, calculated to discourage public support and to cause a falling off in the subscriptions and voluntary contributions which have been so liberally contributed to its resources since H.R.H. the Prince of Wales accepted the office of president.

